# Seasonal effects on egg production and level of paternity in a natural population of a simultaneous hermaphrodite snail

**DOI:** 10.1002/ece3.1560

**Published:** 2015-07-03

**Authors:** Ruben Janssen, Bruno Baur

**Affiliations:** Section of Conservation Biology, Department of Environmental Sciences, University of BaselSt. Johanns-Vorstadt 10, CH-4056, Basel, Switzerland

**Keywords:** Gastropod, hibernation, male function, multiple paternity, sperm storage

## Abstract

In a seasonal environment, the suitable time window for females to reproduce is restricted by both environmental conditions and the availability of males. In simultaneous hermaphrodites, which are female and male at the same time, selection on a trait that is solely beneficial for one sexual function cannot occur independently. Therefore, it is assumed that the optimal time window for reproduction is a compromise between the two sexual functions in simultaneous hermaphrodites, mediated by environmental conditions. We examined seasonal patterns of reproduction and the resulting paternity in a natural population of the simultaneously hermaphroditic land snail *Arianta arbustorum*. Adult and premature individuals (snails in a short protandric phase) were collected on four occasions over the entire active season. The snails were allowed to deposit eggs after which we assessed the level of paternity in their hatched offspring. Individuals mated throughout the reproductive season, whereas egg production – the major task of the female function – was restricted to the first half of the season. Snails collected in autumn were allowed to hibernate under laboratory conditions. As a result, we found that premature individuals began to mate late in the reproductive season, but did not start to produce eggs before emerging from hibernation. Our results demonstrate a temporal shift of reproductive activities; the egg production and oviposition occur mainly in the first half of the season, while sperm production and mating occur over the entire season. In subadult and adult snails, sperm obtained from several partners in the second part of the reproductive season are stored during hibernation for the fertilization of eggs in the successive years. These results extend our understanding of the influence of both natural and sexual selection on reproductive strategies in hermaphrodites.

## Introduction

Sexual selection can act at various stages during the course of reproduction, from male–male combat, even before mating is initiated, all the way to parental care of offspring (Jennions and Kokko 2010). Selection that occurs between copulation and fertilization is termed postcopulatory sexual selection. Mechanisms of postcopulatory selection and manipulation can be separated into sperm competition and cryptic female choice (Birkhead and Pizzari 2002). Sperm competition occurs when sperm from two or more males compete for the fertilization of the female’s ova (Parker 1970). The ways in which females are able to bias the fertilization success of males they have copulated with are collectively known as cryptic female choice (Thornhill 1983; Eberhard 1996). Prerequisites for postcopulatory selection to occur are polyandry (females mating more than once), internal fertilization, and, in certain cases, storage of received sperm (Demont et al. 2011).

Polyandry is widespread in nature (Simmons 2005) and frequently results in multiple paternity (several males siring offspring in a female’s clutch or brood). The degree of a male’s paternity can be influenced by competition with sperm from other males and female manipulation. Studies on multiple paternity are numerous (e.g., Baur 1994a; Griffith et al. 2002; Pongratz and Michiels 2003; Johnson and Yund 2007; Uller and Olsson 2008; Coleman and Jones 2011; Pannell and Labouche 2013). However, very few studies have examined temporal aspects of polyandry. Simmons et al. (2007) and Demont et al. (2011) investigated seasonal changes in multiple mating and sperm storage in nonsocial insects. Both studies found seasonal variation in the number of multiply mated females, as well as in the amount of stored sperm from different males, which highlights the importance of incorporating a time component. Seasonal variation of female mating frequency provides opportunities for males to choose an ideal time for mating. For instance, if females mate more often late than early in the season, males can increase their reproductive success by mating as early as possible, that is, when potential sperm competition intensity is lowest. Therefore, temporal variation of polyandry is one of the several important factors that can affect reproductive success.

The aim of this study was to investigate temporal changes in patterns of reproduction and paternity during the course of the activity season in a natural population of a simultaneous hermaphrodite, the land snail *Arianta arbustorum*. Simultaneous hermaphrodites are female and male at the same time. In contrast to a sexual conflict between females and males in gonochorists (e.g., Simmons et al. 2007; Demont et al. 2011 discussed above), there is a sexual conflict between sexual functions within hermaphroditic individuals (Michiels 1998; Anthes et al. 2010; Schärer et al. 2014). Selection on traits that are solely beneficial for one sexual function cannot occur independently of the other sexual function. Therefore, the optimal mating frequency of a hermaphrodite will be the result of a compromise between the two sexes’ interests. Estimates of the level of multiple paternity represent the minimum sperm competition intensity (Engqvist and Reinhold 2005) and the number of genetic mating partners (i.e., the number of mating partners that are represented in the offspring array; Demont et al. 2011). In reciprocally mating hermaphrodites, estimates of the number of mating partners reflect the minimum mating frequency. Consequently, by investigating the level of multiple paternity, it is possible to discover patterns of mating frequency.

The land snail *A. arbustorum* mates repeatedly over several reproductive seasons (Kupfernagel et al. 2010), has internal fertilization, and is capable of storing sperm for long periods (>1 year, Baur 1988). In this otherwise simultaneous hermaphrodite, sperm production precedes egg production by 2–4 weeks, resulting in a short protandric phase (Baur 1984; Luchtel et al. 1997). Consequently, at any given time, a slightly higher proportion of sperm donors are present in the population, intensifying sperm competition. To comprehensively study sperm competition and paternity in a natural population of *A. arbustorum*, sexually mature individuals (termed adults) were sampled on four occasions, together with premature individuals that represent snails in the protandric phase (termed subadults). To cover the snails’ entire activity season, we sampled from early spring, just after arousal from hibernation, to late autumn, just before entering the next hibernation. Sampled individuals were allowed to oviposit, after which we assessed the level of paternity in their offspring. The sampling of subadults allows to investigate the differences in the beginning of reproduction as male (delivery of sperm during copulation) and as female (production of eggs) in this simultaneous hermaphrodite snail. Based on an earlier study (Kupfernagel and Baur 2011), we expect that subadults will produce no or a small number of egg batches in their first reproductive season. If egg batches are produced, we expect a low level of multiple paternity (e.g., eggs fertilized by one sperm donor). To investigate the viability of stored sperm and the influence of hibernation on reproductive output, individuals sampled in autumn were allowed to oviposit twice, before and after experimental hibernation. We expect that the level of multiple paternity is reduced after hibernation, as a result of decreased viability of stored sperm (Baur 1994a).

To our knowledge, this is the first study that examines patterns of multiple paternity in a natural population of a simultaneous hermaphrodite during an entire activity season. We also investigated reproductive traits that are attributable to the female function or to both sexual functions together. By examining the variance of reproductive output of the female function, it is possible to estimate an upper bound of the opportunity for natural selection (Jones 2009). Bateman gradients (measures of the relative strength of sex-specific sexual selection), however, cannot be calculated because that would require information on the mating success and reproductive success of the male and female function of focal snails (Anthes et al. 2010).

It is important to carry out studies on wild populations and compare these findings with existing knowledge gathered by controlled experiments to fully understand reproductive behavior and sexual selection (Simmons et al. 2007; Demont et al. 2011). In our study, we addressed the following questions: (1) How does the reproductive output of this hermaphrodite snail change over the course of an activity season in a natural population? (2) How does the level of multiple paternity in offspring of focal snails change over the course of an activity season? and (3) How do reproductive output and level of multiple paternity change after hibernation?

## Methods

### Study organism

The simultaneously hermaphroditic land snail *Arianta arbustorum* is common in moist habitats throughout northwestern and central Europe (Kerney and Cameron 1979). This species has determinate growth; adult shell breadth is 16–22 mm. Completion of shell growth is indicated by a reflected lip at the shell aperture (Baur and Raboud 1988). Individuals become sexually mature after 2–4 years and live on average 3–4 more years (Baur and Raboud 1988). However, matings involving subadults, that is, premature individuals that have not yet finished shell growth, have been observed in natural populations (Baur 1984; Kupfernagel and Baur 2011). Therefore, completion of shell growth is a reliable indicator for maturity of the female function of the snail, but not for the male function (Haeussler et al. 2014), that is, egg laying occurs only after completion of shell growth, but sperm production and sperm transfer during copulation can occur in subadult snails.

Matings of *A. arbustorum* last 8–18 h and include elaborate courtship behavior with optional dart shooting (Baur 1992; Baminger et al. 2000). It has been shown that one mating per season is enough to fertilize all the eggs produced by an individual (Chen and Baur 1993), yet multiple paternity has been observed in a natural population (grand mean of 5.5 sperm donors contributing to an individual’s egg output of one season; Kupfernagel et al. 2010). Postcopulatory selection is thought to be especially important in this species, as earlier studies have revealed an absence of precopulatory selection based on shell size, degree of relatedness, and mating experience (Baur 1992; Baur and Baur 1997b; Haeussler et al. 2014). Copulation is reciprocal: both snails transfer simultaneously one spermatophore (Haase and Baur 1995). In the field, snails deposit 1–3 clutches consisting of 20–50 eggs each reproductive season (Baur and Raboud 1988; Baur 1990). Reproduction is primarily by cross-fertilization, although self-fertilization has been observed (Chen 1994; Kupfernagel and Baur 2011). Self-fertilization comes with a substantial reduction in reproductive output (number of selfed hatchlings amounts to 1–2% of those produced by cross-fertilization; Chen 1994).

### Study animals and methods

Snails were sampled in an alpine pasture near Gantrisch, 30 km south of Bern, Switzerland (46°42′N, 7°27′E, elevation 1810 m a.s.l.). Snails occur at high density in this population (>5 adults/m^2^; B. Baur, pers. obs.). Individuals were collected on four occasions in 2012: in spring on 25 May, shortly after arousal from hibernation, in early and late summer on 24 June and 4 August (peak and end of egg production period in the field), and in autumn on 5 September, shortly before entering hibernation (hereafter referred to as groups 1, 2, 3, and 4). The sampling dates span the entire period when snails are active at this elevation (Baur and Raboud 1988). For each group, we collected 16–20 adult and 8–9 subadult individuals.

Adult and subadult snails were kept individually in transparent beakers (diameter 6.5 cm, height 8 cm) lined with approximately 2 cm of moist soil in climate chambers at 18°C ± 1°C and a light: dark cycle of 16:8 h. Twice a week, the beakers were cleaned and fresh lettuce was provided as food. At the same time, the beakers were checked for eggs. Eggs were counted and kept in petri dishes (diameter 6.5 cm, height 2 cm) lined with moist paper towel at 18°C ± 1°C. The petri dishes were checked daily for emerging hatchlings, which were removed to prevent egg cannibalism (Baur 1994b). Emerging hatchlings were stored in order of appearance at −85°C for genotyping analysis.

The reproductive output of each mother snail was recorded as the number of egg batches deposited, the number of eggs per batch, hatching success of eggs, and the number of hatchlings produced. For snails from groups 1 and 2, all eggs deposited over a period of 30 days were collected. For snails from groups 3 and 4, all eggs were collected over a period of 45 days, partly compensating the anticipated lower egg production. After 30 or 45 days, mother snails from groups 1 to 3 were frozen at −85°C for later genotyping. However, for data analyses including all four groups, only data obtained in the first 30 days were considered. For data analyses including exclusively groups 3 and 4, reproductive data gathered over 45 days were considered. The shell breadth of all mother snails was measured to the nearest 0.1 mm using vernier callipers.

It has been shown that individuals arousing from hibernation have sperm stored from the previous year(s) (Baur 1988). However, the viability of sperm decreases with time. Sperm that were stored for more than 300 days showed reduced fertilization success compared to newly acquired sperm (Baur 1994a). To investigate the viability of stored sperm, individuals of group 4 were allowed to oviposit in two periods, before and after experimental hibernation (referred to as groups 4A and 4B). The artificial hibernation period (October 2012 to May 2013 at 4°C and darkness) was similar to its length in this natural population (Baur and Raboud 1988). After arousal from hibernation, snails of group 4B were again kept under the conditions described above for 45 days. Deposited eggs and emerging hatchlings were collected and stored as described above. Individuals collected as subadults in autumn completed shell growth within 45 days after arousal from experimental hibernation.

### Genotyping and paternity analysis

Forty hatchlings of each adult mother snail were genotyped, or all hatchlings if a snail had fewer than 40 offspring. If more than 40 hatchlings were available, then 40 were chosen randomly, but in equal proportions from all clutches produced by the mother. Similarly, 20 hatchlings of each subadult mother snail were genotyped. The number of egg batches produced by adult snails was not equal in the four groups (see Results; Table1a). Therefore, the offspring of all mother snails of groups 3, 4A, and 4B were genotyped, but only a subset of offspring of mother snails of groups 1 and 2 was genotyped the offspring of 11 of 16 and 12 of 16 adult snails.

**Table 1:** Shell size, reproductive traits, and the level of multiple paternity in egg batches of adult and subadult snails collected at different times of the reproductive season. Values are mean ± SD, *n* in parentheses

Sampling time	Group 1	Group 2	Group 3	Group 4	Test statistic	*P*
	Spring	Early summer	Late summer	Autumn		
(a) Adults						
Shell breadth (mm)^1^	17.0 ± 0.6 (16)	17.2 ± 0.8 (16)	17.1 ± 0.6 (13)	16.9 ± 0.8 (15)	*F*_3,56_ = 0.719	0.54
Number of egg batches per individual^1^	2.31 ± 0.87 (16)^a^	2.56 ± 0.63 (16)^a^	0.38 ± 0.65 (13)^b^	0.27 ± 0.46 (15)^b^	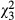 = 54.416	<0.0001
Number of eggs per batch^2^	33.5 ± 8.5 (15)^a^	27.6 ± 7.3 (16)^a^	12.3 ± 2.9 (4)^b^	28.8 ± 10.7 (4)^a^	*F*_3,35_ = 7.679	0.0005
Hatching success (%)^2^	60.9 ± 19.8 (15)^a^	70.7 ± 24.1 (16)^ab^	84.5 ± 11.0 (4)^ab^	92.3 ± 4.2 (4)^b^	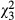 = 10.841	0.0126
Number of hatchlings^2^	48.2 ± 19.8 (15)^a^	50.4 ± 24.3 (16)^a^	12.8 ± 5.7 (4)^b^	26.8 ± 10.6 (4)^ab^	*F*_3,35_ = 4.731	0.0071
Opportunity for selection^1^	0.855^a^	0.988^a^	0.062^b^	0.231^b^	*χ*^2^ = 23.353	<0.0001
Multiple paternity (MP)^3^						
Individuals showing MP (%)	90.9 (11)	91.7 (12)	100 (4)	100 (5)	*χ*^2^ = 1.377	0.71
Egg batches with MP (%)	83.3 ± 32.5 (11)	79.2 ± 31.1 (12)	100 ± 0.0 (4)	100 ± 0.0 (5)	*χ*^2^ = 4.901	0.18
Level of multiple paternity^3^						
Total number of fathers	4.0 ± 2.3 (11)	3.8 ± 1.4 (12)	4.0 ± 0.8 (4)	4.2 ± 1.3 (5)	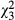 = 0.209	0.98
Minimum number of fathers	2.6 ± 0.8 (11)	3.1 ± 1.4 (12)	2.5 ± 1.0 (4)	3.0 ± 0.0 (5)	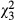 = 0.622	0.89
(b) Subadults						
Shell breadth (mm)^1^	17.6 ± 0.8 (8)	17.0 ± 0.6 (8)	16.9 ± 0.7 (8)	16.7 ± 0.7 (9)	*F*_3,29_ = 2.182	0.11
Number of egg batches per individual^1^	0	0	0.25 ± 0.71 (8)	0.22 ± 0.44 (9)	–	–
Number of eggs per batch^2^	–	–	17 (1)	26.5 ± 6.4 (2)	–	–
Hatching success (%)^2^	–	–	82.4 (1)	100 ± 0.0 (2)	–	–
Number of hatchlings^2^	–	–	28 (1)	26.5 ± 6.4 (2)	–	–
Multiple paternity (MP)^3^						
Individuals showing MP (%)	–	–	100 (1)	100 (2)	–	–
Egg batches with MP (%)	–	–	100 (1)	100 (2)	–	–
Level of multiple paternity^3^						
Total number of fathers	–	–	7 (1)	4.0 ± 2.8 (2)	–	–
Minimum number of fathers	–	–	2 (1)	2.5 ± 0.7 (2)	–	–

–, means no data.

Different small letters indicate significant differences between groups (*P* < 0.05).

1 Includes individuals that did not produce any egg batches.

2 Only individuals that produced eggs.

3 Paternity based on entire egg production per individual.

DNA was extracted using the DNeasy 96 Blood and Tissue Kit protocol from Qiagen AG (2006). Tissue of entire hatchlings was used, whereas a piece of foot tissue (20–30 mg) was cut from adult snails. Primer pairs for six polymorphic microsatellite loci were used (10044, 14477, and 16989 developed by Ecogenics GmbH, Schlieren, Switzerland; Arar9, Arar20, and Arar38 developed by Savannah River Ecology Lab, University of Georgia, U.S.A.; primer sequences are listed in the supplementary table S1). The exclusion probability for the six microsatellites combined was 0.96 (GERUD), considering that the mother was always known.

The volume of 10 *μ*L PCR mixture (HotStarTaq Mastermix Kit, Qiagen AG 2010) comprised of 5–15 ng DNA (3 *μ*L). PCR mixtures were heated at 95°C for 15 min, followed by 35 cycles of 95°C for 30 sec, 56°C/60°C (primer-specific) for 30 sec, and 72°C for 30 sec. The PCR finished with 8 min at 72°C. A QIAxcel DNA High-Resolution Kit (Qiagen AG 2011) for the QIAxcel system (Qiagen AG) was used for separation of the microsatellite allele fragments. For separation, we applied an OL700 method (sample injection 8 KV for 20 sec, separation 3 KV for 820 sec). Homo- or heterozygosity and fragment length were determined for each individual using Screengel software version 1.0 (Qiagen AG). Fragment lengths were determined with size markers (QX DNA Size Marker 25–500 bp, Qiagen AG). Microsatellite characteristics were examined using GenAlEx version 6.5 (Peakall and Smouse 2012), a Microsoft Excel add-on. Statistical power of the paternity analysis was assessed by calculating the probability of detecting multiple paternity using the software PrDM (Neff and Pitcher 2002).

Paternal assignment to the mother–progeny arrays was carried out using the programs GERUD version 2.0 (Jones 2005) and COLONY version 2.0 (Wang 2004). GERUD employs multilocus progeny arrays and a known parental genotype to find the minimum number of complementing parental genotypes. Therefore, GERUD was used to estimate the minimum number of sperm donors matching the mother–offspring data. COLONY is a maximum likelihood program that provides a most probable estimate of the number of paternal genotypes, including one in which every offspring is assigned to a candidate sperm donor or a reconstructed paternal genotype. As *A. arbustorum* is a simultaneous hermaphrodite, the maternal genotypes represent the candidate paternal genotypes. We considered all adult and subadult snails as the genetic pool of candidate sperm donors (*n* = 88). Consequently, using GERUD and COLONY, we obtained an estimate of the minimum and the total number of sperm donors siring offspring with every mother snail.

Null alleles can cause errors in estimates of parentage assignment (Dakin and Avise 2004). In a preliminary analysis, 24 individuals of the Gantrisch population (none used as mother snails in this study) were genotyped using primer pairs for 25 microsatellite loci. Six polymorphic microsatellites that showed no sign of null alleles were selected for this study (Table S1).

### Statistical analysis

Reproductive traits and the level of multiple paternity were tested for the effects of sampling date (groups 1, 2, 3, 4A, and 4B) and growth status (adult/subadult). Shell size was included as a covariant in all analyses. All response variables were tested for normality using the Shapiro–Wilk normality test. Adult snails were exclusively considered in tests comparing the number of eggs per batch, hatching success, the number of hatchlings produced, proportion of multiple paternity in individuals and egg batches, and the level of multiple paternity; subadult individuals were excluded due to low egg production (see Result Table1b). Snails Ga78 (group 2) and Ga111 (group 4) both produced one clutch with few big, ellipsoid eggs. These eggs were unfertilized and denote a way in which *A. arbustorum* gets rid of long-standing stored egg mass. The number of egg batches deposited and the number of eggs per batch of both snails were therefore treated as 0.

To examine whether reproductive traits and the level of multiple paternity change in the course of the season, groups 1–4A were compared. Only egg batches deposited within 30 days were considered in groups 3 and 4A, to ensure equal comparison with groups 1 and 2. A one-way ANOVA was used to test for differences in shell size of mother snails. Generalized linear models (GLMs) with Poisson error structure were used to examine whether snails with different sampling date differed in the number of egg batches deposited and the level of paternity. Similarly, ANCOVAs were applied to test whether the number of eggs per batch and the number of hatchlings produced differed among snails with different sampling date. A mixed effect logistic regression (MELR) with binomial error structure was used to test whether hatching success differed among snails (the use of this special case of generalized linear mixed model (GLMM) is recommended for overdispersed binomial data, Warton and Hui 2011). GLMs with binomial error structure were used to test whether the proportion of individuals with multiple paternity in their egg batches differed among snails. Finally, GLMs with binomial error structure were also used to test whether the percentage of egg batches with multiple paternity differed among snails. Tukey’s honest significant differences (TukeyHSD) were applied to obtain pairwise comparisons of means. Each GLM, ANCOVA, and MELR was started with a maximal model including sampling date, growth status, and shell size. Then, stepwise simplification was performed based on Akaike information criterion (AIC) and chi-squared deletion tests to select the minimal adequate model.

To obtain an estimate of the opportunities for selection (Jones 2009), the variance in reproductive success of adult mother snails of groups 1–4A was calculated. The total number of offspring divided by the average reproductive success across all groups gives the standardized reproductive success of each adult snail. Variance in standardized reproductive success (*I*_f_) denotes an upper bound of the opportunity for natural selection on the female function. Fligner–Killeen tests for homogeneity of variance were used to test whether variance differed between groups.

To examine whether reproductive traits changed following hibernation, group 4A was compared with group 4B. Only snails with data in both periods were considered. As identical snails were considered twice, a GLMM with Poisson error structure and snail identity as a random factor was used to test whether the number of egg clutches deposited differed before and after hibernation. Further reproductive traits could not be compared, due to the low number of individuals that deposited egg batches before hibernation (adults: *n* = 3, subadults: *n* = 2). In addition, two other comparisons were made to examine the effect of hibernation on reproductive traits and the level of multiple paternity. Firstly, reproductive output and the level of multiple paternity were compared between adult and previously subadult snails of group 4B. Statistical tests were applied as described for groups 1–4A, except that the percentage of egg batches with multiple paternity was tested with a GLM with quasibinomial error structure. Secondly, to compare egg laying after hibernation in the field with hibernation under experimental conditions, the number of egg batches deposited by adult snails of groups 1 and 4B was compared as described above.

All statistical analyses were carried out with the statistical computing environment R (R Development Core Team 2013), using the packages “lme4” (Bates et al. 2014), “lattice” (Sarkar 2008), and “multcomp” (Hothorn et al. 2008).

## Results

### Seasonal patterns in reproductive traits

**Figure 1:**
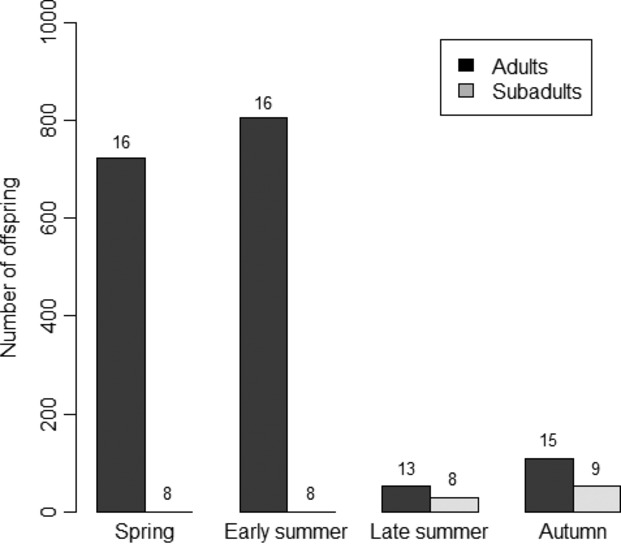
The total number of offspring produced by all mother snails over 30 days in the four seasonal groups. Adult mothers in black, subadult mothers in light gray, and *n* above bars.

The reproductive output of adult and subadult snails collected in spring, early summer, late summer, and autumn was compared. The proportion of adult snails that produced eggs varied in the course of the activity season, being highest in the groups of snails collected in spring and early summer. Considering all adult snails, the mean number of egg batches deposited by snails collected in spring and early summer was higher than the number produced by snails collected in late summer and autumn (Table1a). When only snails that laid eggs were considered, there was a slight tendency that snails collected in spring and early summer produced more eggs than the snails collected later in the season (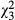 = 6.596; *P* = 0.0860). Egg batches deposited by adult snails also differed in size over the season (Table1a). Snails collected in late summer produced fewer eggs per batch than those of the other groups. Furthermore, hatching success of eggs from adult snails changed over the season. In autumn, hatching success of eggs was higher than hatching success of eggs in spring, while hatching success did not differ between eggs produced in spring, early or late summer (Table1a). The number of hatchlings emerging from eggs changed over the season as well. Snails collected in late summer produced fewer hatchlings than those collected in spring or early summer, whereas the number of hatchlings produced by snails collected in autumn did not differ from the other groups (Table1a). The total number of offspring produced by mother snails as a group differed over the season (Fig.1). There was a significant difference in the opportunity for natural selection on the female function (*I*_f_) between the seasonal groups (Table1a). Shell size of mother snails did not affect any reproductive trait, neither in adults nor in subadults (stepwise chi-square-deletion tests *P* ≥ 0.32).

The proportion of subadult snails that produced eggs did not differ across the season. However, there were too few subadult snails that produced eggs to allow statistical analyses of further reproductive traits (Table1b).

### Reproductive traits before and after hibernation

Snails collected in autumn were allowed to hibernate for 6.5 months, and their reproductive output over 45 days before and after hibernation was compared. A larger proportion of both adult and previously subadult snails produced eggs after hibernation compared to before. Consequently, the mean number of egg batches deposited after hibernation was higher compared to before (Table2a and b).

**Table 2:** Shell size, reproductive traits, and the level of multiple paternity in egg batches of adult and subadult snails collected in autumn before and after experimental hibernation. Values are mean ± SD, *n* in parentheses

	Before hibernation	After hibernation	Test statistic	*P*
(a) Reproductive traits adults				
Shell breadth (mm)^1^	17.1 ± 0.8 (10)	17.1 ± 0.8 (10)	–	–
Number of egg batches per individual^1^	0.40 ± 0.70 (10)	2.30 ± 0.82 (10)	*χ*^2^=11.007	0.0009
Number of eggs per batch^2^	24.4 ± 11.6 (3)	24.7 ± 8.9 (3)	–	–
(b) Reproductive traits subadults				
Shell breadth (mm)^1^	16.7 ± 0.7 (8)	16.7 ± 0.7 (8)	–	–
Number of egg batches per individual^1^	0.25 ± 0.46 (8)	3.00 ± 1.07 (8)	*χ*^2^=13.076	0.0003
Number of eggs per batch^2^	26.5 ± 6.4 (2)	22.9 ± 9.1 (2)	–	–

	Adults	Subadults	Test statistic	*P*
(c) Adults and subadults after hibernation				
Shell breadth (mm)^1^	17.1 ± 0.8 (10)	16.7 ± 0.7 (8)	*F*_1,15_ = 1.265	0.28
Number of egg batches per individual^1^	2.30 ± 0.82 (10)	3.00 ± 1.07 (8)	*χ*^2^ = 0.482	0.49
Number of eggs per batch^2^	24.7 ± 8.9 (9)	22.1 ± 5.2 (8)	*F*_1,15_ = 0.499	0.49
Hatching success (%)^2^	81.1 ± 14.6 (9)	70.3 ± 18.5 (8)	*χ*^2^ = 2.086	0.15
Number of hatchlings^2^	46.9 ± 19.6 (9)	48.0 ± 22.1 (8)	*F*_1,15_ = 0.012	0.91
Multiple paternity (MP)^3^				
Individuals showing MP (%)	88.9 (9)	75.0 (8)	*χ*^2^ = 0.568	0.45
Egg batches with MP (%)	79.6 ± 35.1 (9)	68.8 ± 45.8 (8)	*F*_1,15_ = 0.627	0.44
Level of multiple paternity^3^				
Total number of fathers	3.3 ± 1.2 (9)	3.0 ± 1.2 (8)	*χ*^2^ = 0.149	0.70
Minimum number of fathers	2.8 ± 1.0 (9)	2.4 ± 0.7 (8)	*χ*^2^ = 0.267	0.61

–, means no data.

1 Includes individuals that did not produce any egg batches.

2 Only individuals that produced eggs.

3 Paternity based on entire egg production per individual.

After hibernation, similar proportions of adult and previously subadult individuals produced eggs. Furthermore, adult and previously subadult individuals did not differ in number of egg batches deposited, batch size, hatching success of eggs, and number of hatchlings produced (Table2c).

To compare reproductive output after hibernation in the field with that in the laboratory, adult snails that were collected in spring and adult snails that were collected in the previous autumn were considered. The number of egg batches deposited did not differ between these groups (mean ± SD; spring: 2.31 ± 0.87; autumn and hibernation: 1.60 ± 0.97; *χ*^2^ = 1.584; *P* = 0.21).

### Level of multiple paternity

In total, 56 adult and 32 subadult snails were genotyped (13 adults and 1 subadult died during the study), together with 1436 hatchlings (of 1954; 73.5%) from 38 adult mothers, and 204 hatchlings (of 465; 43.9%) from nine subadult mothers. There was no amplified product with any of the six primers in one subadult snail and six offspring (of 1728 samples; 0.4%), and it was assumed that DNA extraction failed. PCR amplification of 251 loci of 225 individuals failed. This corresponds to 2.4% of all loci genotyped. As GERUD is not able to deal with null alleles, individuals with one or more missing alleles were omitted in the GERUD estimate, which resulted in 87.0% of all genotyped offspring being used. All genotyped offspring could be used in the COLONY estimate.

Multiple paternity was found in 38 of 41 progenies of adult snails (92.7%; all seasonal groups considered), in 3 of 3 progenies of subadult snails, and in 6 of 8 progenies of previously subadult snails (75.0%). The proportion of adult snails that produced egg batches with multiple paternity did not differ across the season (Table1a). Considering single egg batches of adult snails, there was no difference across the season in the proportion of egg batches with multiple paternity (Table1a). Statistical power of multiple paternity analyses was high: The probability of detecting multiple paternity was in all cases >0.97 when assuming skewed paternities (PrDM).

**Figure 2:**
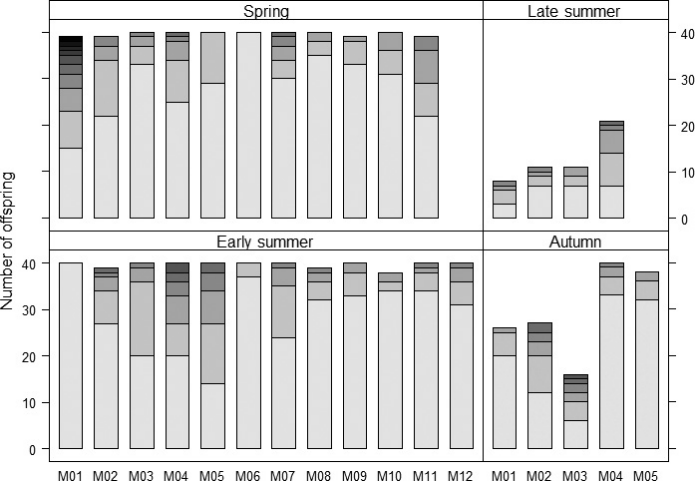
Paternity shares of different sperm donors in progenies of mother snails in the four seasonal groups by COLONY. Similar shades of gray do not correspond to identical sperm donors.

The minimum number of sperm donors by GERUD ranged from 1 to 6 contributing sperm donors to reproducing adult snails (grand mean of groups 1–4: 2.8). There was no difference across the season in the mean level of multiple paternity by GERUD (Table1a). The range of contributing donors narrowed from 1 to 6 in snails collected in spring to 3–3 (no variance) in snails collected in autumn. There was a tendency toward a difference in standardized variance between the groups (*χ*^*2*^ = 6.954; df = 3; *P* = 0.0734; pairwise comparison groups 2 and 4A: *χ*^*2*^ = 5.243; df = 1; *P* = 0.0220; other pairwise comparisons: *P* ≥ 0.10). The total number of sperm donors by COLONY (error rate 0.005) ranged from 1 to 10 contributing sperm donors to reproducing adult snails (grand mean of groups 1–4: 4.0). Again, there was no difference across the season in the mean level of multiple paternity by COLONY (Table1a). Analyses by COLONY using more conservative error rates (0.01 and 0.02) resulted in a smaller range of contributing donors in both cases (1–7). However, means of the level of multiple paternity with more conservative error rates also showed no difference over the season (GLM: 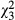
* *≤ 1.876; *P* ≥ 0.60). The range of contributing donors narrowed from 1 to 10 in adult snails collected in spring to 3–6 in adult snails collected in autumn, but there was no difference in variance (*χ*^*2*^ = 1.741; df = 3; *P* = 0.63). COLONY provides a maximum likelihood estimate of the paternity share of each contributing sperm donor to a progeny (Fig.2).

Considering snails after hibernation in the laboratory, adult and previously subadult snails did not differ in the proportion of individuals that produced egg batches with multiple paternity (Table2c). Adult and previously subadult individuals did not differ in the proportion of single egg batches with multiple paternity (Table2c). After experimental hibernation, the level of multiple paternity in the offspring of previously subadult snails was not different from that of the adult snails collected at the same time (Table2c). In snails that deposited egg batches before and after hibernation (adults: *n* = 3, subadults: *n* = 2), the relative contribution of sperm donors to egg batches did not change, except in one subadult individual.

## Discussion

### Seasonal patterns in reproductive traits

We found seasonal variation in egg production, which is reproduction through the female function in simultaneous hermaphrodites (Schärer 2009). The majority of egg laying took place in the first half of the season. In several land snails, including *A. arbustorum*, winter mortality of juveniles is negatively correlated with their shell size (Terhivuo 1978; Baur and Baur 1991). It is therefore beneficial for snails to deposit eggs early in the season so that offspring have time for growth before hibernation (Baur 1990). *A. arbustorum* cannot hibernate in the egg stage. Egg production in land snails is influenced by the photoperiod; shortening day length results in a reduction or complete stop of egg laying (Gomot de Vaufleury 2001). Earlier studies on *A. arbustorum* reported a decrease in batch size throughout the season (Baur 1990; Baur and Baur 1997a). Our finding that individuals produced fewer egg batches in late summer and autumn than in spring and early summer agrees with this finding. Offspring that hatch late in the season have a greater chance of survival if they are larger, which in turn depends on egg size and nutrient content (Baur and Baur 1997a). However, the relationships between time of egg laying, egg size, and nutrient content may vary across populations depending on local selection pressures (Baur and Baur 1997a). An additional reason for reducing egg output might be the fact that egg production is energetically costly. Locher and Baur (2000) showed that resource allocation (measured as dry weight or nitrogen content) was always highly female biased; the average proportion of resources devoted to the male function (sperm and spermatophore) never exceeded 5%. It might therefore be too costly to maintain egg laying until short before hibernation. Snails likely reduce their egg output to save energy for hibernation, and to avoid the production of offspring which immediately face a cold period.

Individuals of *A. arbustorum* that were collected at the end of the active season and subsequently hibernated in the laboratory had a similar reproductive output in the following spring as snails that were collected immediately after hibernation in the field. This indicates that the lower output in autumn, that is, before hibernation, was likely not a result of lack of sperm, but rather due to the imminent winter. Clutch size was lowest in late summer, the hottest period of the season. Land snail activity is generally low during periods of high temperature (Cook 2001). Furthermore, it has been shown that egg mortality increases with increasing temperature (Baur and Baur 1993); thus, it is not beneficial to deposit eggs during hot periods. The finding that the opportunity for natural selection on the female function was larger in the first half of the season than in the second half likely reflects a combination of temperature restrictions, saving energy for hibernation and producing offspring at a suitable time (discussed above).

Hatching success of eggs was lowest in spring and increased over the season. This result most probably reflects the reduced viability of sperm stored from matings in the previous year(s). In *A. arbustorum*, sperm received from matings in the previous season contributed less to fertilization of eggs than newly received sperm (Baur 1994a). In the present study, snails collected in spring had just aroused from hibernation; therefore, the sperm that fertilized their eggs were obtained in the previous season(s). In contrast, snails collected in summer and autumn produced egg clutches fertilized with new sperm gathered throughout the ongoing season. This group showed a significantly higher hatching success of eggs.

### Seasonal patterns of multiple paternity

Multiple paternity was found in progenies of snails from all seasonal groups. Furthermore, our results showed that multiple paternities were always highly skewed; the majority of eggs in a clutch were fertilized by one or two donors (Fig.2). However, the number of snails and egg batches showing multiple paternity, as well as the level of paternity in offspring, did not differ across the season. Although the level of paternity in progenies consistently averaged four across the season, the skew toward one or two main sperm donors suggests differences in ejaculate quality or a selective storage of sperm from different mating partners. While these estimates of skew are informative, they should be interpreted with caution, as assigning each offspring to a specific sperm donor requires making assumptions about combining sperm haplotypes into diploid paternal genotypes (Johnson and Yund 2007). To which extent the higher fertilization success of certain sperm is a result of higher competitive ability of certain ejaculates or selection by the recipient (cryptic female choice) remains to be examined. It has been shown, however, that reproductive success can be influenced by several factors in simultaneously hermaphroditic snails, for example, by the mating order (Baur 1994a; Rogers and Chase 2002; Garefalaki et al. 2010), by an increased activity resulting in more copulations (Minoretti et al. 2011), or by manipulating sperm uptake through the shooting of darts into the soft body of the recipient (Rogers and Chase 2002; Nakadera and Koene 2013).

Multiple mating has clear advantages for males in gonochoristic species, as it increases fertilization success (Bateman 1948), and the same strategy is beneficial for the male function in hermaphrodites (Charnov 1979). Multiple paternity is a result of multiple mating. However, if one mating is enough to fertilize all the eggs of an individual snail in one season (Chen and Baur 1993), there have to be benefits of multiple mating for the female function too, as costs of mating can be substantial (Daly 1978). In *A. arbustorum*, repeated mating increases egg production and may serve as a hedge against unsuccessful matings (Baur and Baur 1992; Chen and Baur 1993). In addition, multiple mating potentially allows for sperm competition between ejaculates, which results in good sperm competition ability in the offspring (Keller and Reeve 1995), and multiple paternity implies high genetic diversity among offspring (Jennions and Petrie 2000). In the present study, recurrent multiple paternity was found across the season, which suggests, in accordance with earlier studies, that both sexual functions benefit from multiple mating in *A. arbustorum*.

There are several, mutually not exclusive scenarios to explain the absence of seasonal variation in multiple paternity. As a first possibility, it may be that after a certain number of successful matings, the sperm storage organ (termed spermatheca) is saturated. In the present study, sperm stored from on average four mating partners would indicate saturation. Whether this explanation is likely remains to be confirmed. However, Beese and Baur (2006) have shown in *A. arbustorum* that already 45% of the spermatheca’s volume is filled after one successful copulation. Sperm displacement, that is, active removal of sperm from previous mates by the current mating partner, is unlikely in this species. Many stylommatophoran gastropods, including *A. arbustorum*, transfer sperm to their partner in a spermatophore (Gómez 2001). When spermatophore transfer is successful, sperm have to escape digestion and migrate up the spermoviduct to the sperm storage organ (Gómez 2001). The sperm storage organ is physically not accessible for mating partners. In contrast to studies on sperm displacement in insects (e.g., Córdoba-Aguilar et al. 2003; Xu and Wang 2010) or a flatworm (Marie-Orleach et al. 2014), there is, to our knowledge, no information on sperm displacement in stylommatophoran land snails.

Secondly, the level of multiple paternity in progenies estimated for every snapshot moment of the season reflects the minimum mating frequency. Consequently, snails had mated four times prior to each snapshot moment. However, the fact that stored sperm can be viable for more than 1 year (Baur 1988) might misrepresent the level of multiple paternity found. Mating activity is very low or not occurring at all in hot periods during summer (when activity of land snails is generally low; Cook 2001), but, probably due to the longevity of stored sperm, we found no decrease in the level of paternity in offspring. Neither did we find any increase toward the end of the season, when mating activity increases again (B. Baur, pers. obs., and subadults in this study, see below). From an evolutionary point of view, the long-term storage of viable sperm has several advantages (Orr and Zuk 2012 and references therein).

As a third possibility, variability of individual morphology might explain the variation in the level of multiple paternity found. The spermatheca of *A. arbustorum* shows great variability in morphology and number of tubuli, that is, blind ending ducts for storage (2–9 tubuli; Bojat et al. 2001). The paternity variation found might simply reflect each snail’s ability to store sperm segregated. Spermathecal morphology is also variable in many other snail species (e.g., 4–19 tubuli in *Cornu aspersum*; Koemtzopoulos and Staikou 2007; 3–5 tubuli in *Helix pomatia*; Evanno and Madec 2007). Whether a more complex spermatheca can store more sperm is still unresolved; Bojat and Haase (2002) found a positive correlation between the number of tubuli and the amount of sperm in the spermatheca of *A. arbustorum*, while Beese and Baur (2006) found no relationship. To the best of our knowledge, a correlation between the number of spermathecal tubuli and level of multiple paternity in offspring has so far not been found in land snails.

Earlier studies on *A. arbustorum* found a similar or slightly higher number of contributing donors to all offspring produced by snails in one season (Kupfernagel et al. 2010; Kupfernagel and Baur 2011). However, our study only considered egg batches produced within 30 days, and the number of donors should therefore be considered as a minimum estimate. Generally, field data on multiple paternity probably underestimate the actual mating frequency (Demont et al. 2011), for example, as a result of unsuccessful sperm transfer during copulation (Chen and Baur 1993). Under semi-natural conditions, individuals of *A. arbustorum* mated between zero and three times within 58 days (Minoretti et al. 2011), which coincides well with the mating frequency (i.e., level of multiple paternity) that was found in the present study. There was more variance among snails in the minimum number of contributing sperm donors in early summer than in autumn. This suggests less variation between snails in mating frequency toward the end of the season, which supports the hypothesis presented above that after a certain number of matings, the spermatheca is saturated.

### Subadult reproduction

A fully developed spermatheca, which is an important prerequisite for reproduction through the female function, is already present in subadults (Baminger and Haase 2002). However, our study showed that subadults deposit less egg batches than adults throughout the season. Subadults may still need to invest resources into growth (Kupfernagel and Baur 2011).

Surprisingly, a high level of multiple paternity was found in offspring of previous subadults that hibernated in the laboratory. After hibernation, these previously subadult snails did not differ from adults in any of the female reproductive traits examined, and the level of multiple paternity in offspring did not differ from that in offspring of adults. These results indicate that many subadults have already mated several times by the end of the season, are able to successfully store the received sperm and use it for fertilization after hibernation. Hardly any subadults deposited eggs in the period prior to hibernation, but they successfully mated at least three times, on average, as indicated by the level of paternity found. In the wild, sperm received from subadult mating partners are used in the same frequency for the fertilization of eggs as sperm from adult partners (Kupfernagel and Baur 2011).

## Conclusions

To summarize, our results indicate that the reproductive behavior of *A. arbustorum* is optimized to seasonal characteristics and intra-individual tradeoffs. By collecting individuals on four occasions, we gathered snapshot information at four distinct behavioral phases of the reproductive season. There seems to be a temporal shift in reproductive activities from oviposition and mating in the first half of the season to mainly mating in the second half. The first half of the season is used to produce as many offspring as possible, that is, to maximize female reproductive output. In the last months of the season, no further eggs are produced but mating activity is still maintained. The main function of the male function is to donate sperm, which can be carried out throughout the season (whenever climatic conditions allow). Mating is the necessary realization of donating sperm. However, mating is beneficial for both sexual functions. It is therefore difficult to speculate on which sexual function drives the mating behavior. Nevertheless, transferring a spermatophore is less energetically costly than laying eggs (Locher and Baur 2000). Sperm acquired in matings can be stored until the next reproductive season starts. Subadult snails seem to be already reproductively active through the male function before complete sexual maturity.

## Supporting Information

**Table S1.** Summary statistics of six microsatellite loci for *A. arbustorum* of Gantrisch population based on 86 mother snails.
