# Supplementary material for: Seasonal effects on egg production and level of paternity in a natural population of a simultaneous hermaphrodite snail
Source: Ecol Evol. 2015 Jul 3;5(14):2916–28. doi: 10.1002/ece3.1560 (PMC4541995; doi:10.1002/ece3.1560)
Supplement: Supplementary file 1 [file ece30005-2916-sd1.doc]

Supplementary table S1. Summary statistics of six microsatellite loci for Arianta arbustorum of Gantrisch population based on 86 mother snails.	
Locus	Primer sequence (5'–3')	Ta (°C)	N	HO	HE	Exclusion probability*	
Arar9	F: CAGTCGGGCGTCATCAGGATGTACCCTCCCATTTGC
R: CACTTTCAACTTCCCGAGCC	60	7	0.62	0.82	0.63	
Arar20	F: CAGTCGGGCGTCATCATGTTATGGTCTATCCAGCGCC
R: CTGTGTGTTGCCGGATGC	60	7	0.55	0.65	0.42	
Arar38	F: CAGTCGGGCGTCATCAGATGATACAGACCGGATTACGC
R: TGGAGATACGGCTGACTTGC	60	7	0.53	0.68	0.47	
10044	F: ACGACGAGGAAGATGAGGAG
R: TTACATTTGTCACTGTCTTGTTATTC	56	4	0.45	0.46	0.26	
14477	F: GGTAAGGGTGTGAGCTTCATTATC
R: ATGAAAACCCGGTCTCCCTG	56	5	0.50	0.67	0.39	
16989	F: GAGATCCATTGCAGCCAACC
R: GATCCAAATGTGAGCCACGG	56	3	0.30	0.29	0.15	
All loci (one parent known)
 	0.96	
F, forward primer; R, reverse primer; Ta, optimal annealing temperature; N, number of alleles at locus; HO, observed heterozygosity; HE, expected heterozygosity;
* Exclusion probability estimates by GERUD.	
